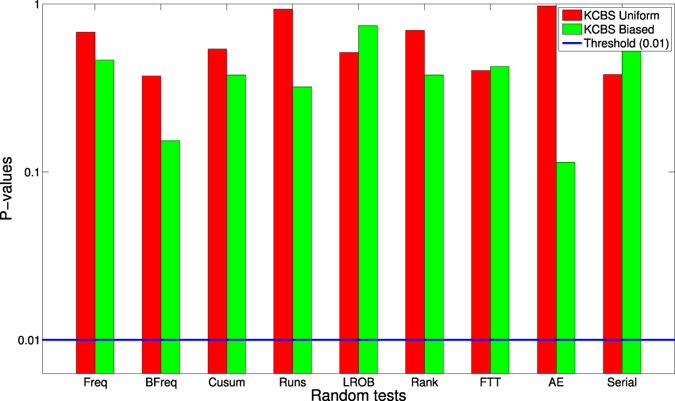# Corrigendum: Experimental Certification of Random Numbers via Quantum Contextuality

**DOI:** 10.1038/srep46927

**Published:** 2018-02-02

**Authors:** Mark Um, Xiang Zhang, Junhua Zhang, Ye Wang, Yangchao Shen, D.-L. Deng, Lu-Ming Duan, Kihwan Kim

Scientific Reports 3: Article number: 1627; 10.1038/srep01627 published online: 04 09 2013; updated: 02 02 2018.

The original version of this Article contained an error in the spelling of the author Yangchao Shen, which was incorrectly given as Shen Yangchao.

This error has now been corrected in the PDF and HTML versions of the Article.

In addition, the 〈*V*_*i*_*V*_*j*_〉 terms in Table 1 were omitted from the calculation of 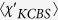 in Equation 4. Therefore, in Table 1,
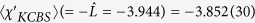


should read:
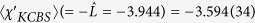


As a result, in the Abstract,

“In our experiment, we generate 1 × 10^5^ random numbers that are guaranteed to have 5.2 × 10^4^ bits of minimum entropy with a 99% confidence level.”

should read:

“In our experiment, we generate 1 × 10^5^ random numbers that are guaranteed to have 2.4 × 10^4^ bits of minimum entropy with a 99% confidence level.”

In the Results section, under subheading ‘Random number results’,

“As shown in Table 1, we observe the expectation 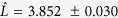, implying the min-entropy 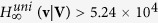 with 99% confidence. Note that the other confidence level *δ* does not have any noticable influence on the bound of min-entropy. Here we used the thresholds of KCBS violations 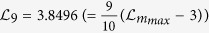.”

should read:

“As shown in Table 1, we observe the expectation 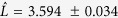, implying the min-entropy 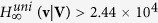 with 99% confidence. Note that the other confidence level *δ* does not have any noticeable influence on the bound of min-entropy. Here we used the thresholds of KCBS violations 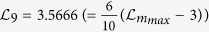.”

In the title of Table 1,

“Our experimental test clearly shows the violation of the extended inequality (3) with 31 σ”

should read:

“Our experimental test clearly shows the violation of the extended inequality (3) with 18 σ”

Moreover, the presented data for the biased choice of measurement settings does not show the net randomness after including the terms 〈*V*_*i*_*V*_*j*_〉 in Table 1 for the 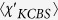 in Equation 4. it is necessary to double the total experimental round as *n* = 2 × 10^5^ with the new biased distribution parameter *α* = 12 in order to observe the net randomness. Therefore, the contents of the paper related to the biased choice of measurement settings should be corrected as follows.

In the Results section, under subheading ‘Random number results’,

“We also generate random bits with a biased choice of measurement settings, where *P* (*V*_1_) = 1 − 4*q*, *P* (*V*_2_) = *P* (*V*_3_) = *P* (*V*_4_) = *P* (*V*_5_) = *q*, and *q *= *αn*^−1/2^ with *α *= 6 and *n *= 10^5^. We observe basically the same behavior of the min-entropy for the generated stream except for a slightly smaller bound due to the non-uniform setting. We get the min-entropy bound 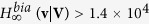 from 1 × 10^5^ rounds with violation of 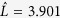. For the biased choice of measurement settings, the output entropy (1.35 × 10^4^) exceeds the input entropy (1.14 × 10^4^), and we obtain 2.1 × 10^3^ net random bits.”

should read:

“We also generate random bits with a biased choice of measurement settings, where *P* (*V*_1_) = 1 − 4*q*, *P* (*V*_2_) = *P* (*V*_3_) = *P* (*V*_4_) = *P* (*V*_5_) = *q*, and *q* = *αn*^−1/2^ with *α* = 12 and *n* = 2 × 10^5^. We observe basically the same behavior of the min-entropy for the generated stream except for a slightly smaller bound due to the non-uniform setting. We get the min-entropy bound 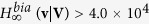 from 2 × 10^5^ rounds with violation of 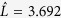. For the biased choice of measurement settings, the output entropy (3.95 × 10^4^) exceeds the input entropy (3.28 × 10^4^), and we obtain 6.8 × 10^3^ net random bits.”

In the legend of Figure 4,

“(a)(c)The min-entropy 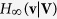 (8) depending on the number of trials for (a) an uniform distribution of measurement settings *P*(*V*_*i*_) = 1/5 and (c) a biased distribution with *P* (*V*_1_) = 1 − 4*q*, *P* (*V*_2_) = *P* (*V*_3_) = *P* (*V*_4_) = *P* (*V*_5_) = *q*, where *q* = 6(100000)^−1/2^ with the probablity of errors 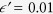 and 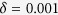. The min-entropies 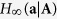 (8) are bounded by the relation of the violation 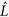 of the KCBS inequality (8), where we set the 10 intervals of 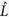 between 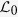 and 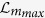. The min-entropies are linearly increasing as the number of trial increases and the slopes are basically dependent on the thresholds of the intervals 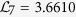 (blue),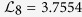 (green), 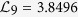 (yellow), and 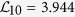 (red).”

should read:

“(a)(c)The min-entropy 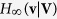 (8) depending on the number of trials for (a) an uniform distribution of measurement settings *P*(*V*_*i*_) = 1/5 and (c) a biased distribution with *P* (*V*_1_) = 1 − 4*q*, *P* (*V*_2_) = *P* (*V*_3_) = *P* (*V*_4_) = *P* (*V*_5_) = *q*, where *q* = 12(200000)^−1/2^ with the probability of errors 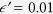 and 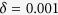. The min-entropies 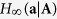 (8) are bounded by the relation of the violation 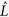 of the KCBS inequality (8), where we set the 10 intervals of 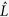 between 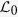 and 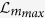. The min-entropies are linearly increasing as the number of trial increases and the slopes are basically dependent on the thresholds of the intervals.”

Figures 4 and 5 based on the corrections of the 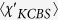 for the uniform distribution and the new data for the biased choice of measurement setting are shown below as Figures 1 and 2, respectively.

In addition, this Article contains typographical errors in the Results section, under subheading ‘The KCBS inequality’.

“Here |*v*_1_〉 = |1〉, |*v*_2_〉 = |2〉, |*v*_3_〉 = *R*_1_ (*γ*, 0) |*v*_1_〉, |*v*_4_〉 = *R*_2_ (*γ*, 0) |*v*_2_〉, |*v*_5_〉 = *R*_1_ (*γ*, 0) |*v*_3_〉 and 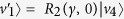, where *γ* = 51.83° and *R*_1,2_ denote the rotation operations between |1〉 to |3〉 and between |2〉 to |3〉, respectively.”

should read:

“Here |*v*_1_〉 = |1〉, |*v*_2_〉 = |2〉, |*v*_3_〉 = *R*_1_^−1^ (*γ*, 0) |*v*_1_〉, |*v*_4_〉 = *R*_1_^−1^ (*γ*, 0) *R*_2_ (*γ*, 0) |*v*_2_〉, |*v*_5_〉 = *R*_1_^−1^ (*γ*, 0) |*v*_3_〉*R*_2_^−1^ (*γ*, 0) |*v*_3_〉 and 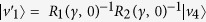, where *γ* = 103.68° and *R*_1,2_ denote the rotation operations between |1〉 to |3〉 and between |2〉 to |3〉, respectively.”

In the legend of Figure 1,

“(b) The pulse sequence to prepare 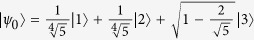. Here, *R*_1_ and *R*_2_ represent the coherent rotations between |1〉 to |3〉 and between |2〉 to |3〉, respectively, where *θ* = 41.97° and *ϕ* = 64.09°. The sequence starts from |3〉 state (black filled circle) after optical pumping. (c)–(g) The pulse sequences for the measurement configurations (c) *A*_1_*A*_2_, (d) *A*_2_*A*_3_, (e) *A*_3_*A*_4_, (f) *A*_4_*A*_5_, (g) *A*_5_*A’*_1_, where *γ* = 51.84°.”

should read:

“(b) The pulse sequence to prepare 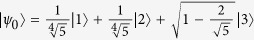. Here, *R*_1_ and *R*_2_ represent the coherent rotations between |1〉 to |3〉 and between |2〉 to |3〉, respectively, where *θ* = 83.94° and *ϕ* = 128.18°. The sequence starts from |3〉 state (black filled circle) after optical pumping. (c)–(g) The pulse sequences for the measurement configurations (c) *A*_1_*A*_2_, (d) *A*_2_*A*_3_, (e) *A*_3_*A*_4_, (f) *A*_4_*A*_5_, (g) *A*_5_*A’*_1_, where *γ* = 103.68°.”

## Figures and Tables

**Figure 1:**
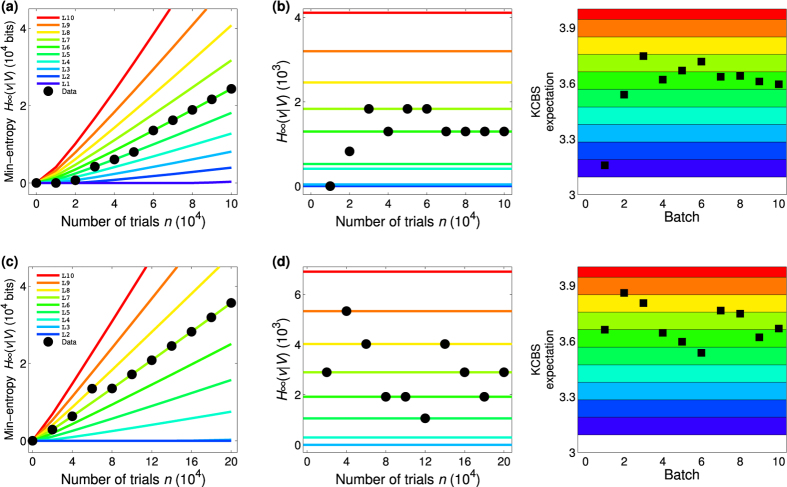


**Figure 2:**